# Hydroxylamine released by nitrifying microorganisms is a precursor for HONO emission from drying soils

**DOI:** 10.1038/s41598-018-20170-1

**Published:** 2018-01-30

**Authors:** M. Ermel, T. Behrendt, R. Oswald, B. Derstroff, D. Wu, S. Hohlmann, C. Stönner, A. Pommerening-Röser, M. Könneke, J. Williams, F. X. Meixner, M. O. Andreae, I. Trebs, M. Sörgel

**Affiliations:** 1 Biogeochemistry Department, Max Planck Institute for Chemistry, P.O. Box 3060, 55020 Mainz, Germany; 2 Institute for Inorganic and Analytical Chemistry, Johannes Gutenberg University Mainz, 55128 Mainz, Germany; 3 Biogeochemical Processes Department, Max Planck Institute for Biogeochemistry, P.O. Box 10 01 64, 07745 Jena, Germany; 4 Atmospheric Chemistry Department, Max Planck Institute for Chemistry, P.O. Box 3060, 55020 Mainz, Germany; 5 Key Laboratory of Agricultural Water Research, Center for Agricultural Resources Research, Institute of Genetic and Developmental Biology, The Chinese Academy of Sciences, 050021 Shijiazhuang, China; 6 Department of Microbiology and Biotechnology, University of Hamburg, Hamburg, Germany; 7 MARUM Center for Marine Environmental Sciences and Department of Geoscience, University of Bremen, P.O. Box 330440, 28334 Bremen, Germany; 8 Scripps Institution of Oceanography, University of California San Diego, San Diego, CA 92093 USA; 9 Present Address: Messer Industriegase GmbH, Messer-Platz 1, 65812 Bad Soden, Germany; 10 Present Address: Key Laboratory of Geographic Information Sciences, Ministry of Education, School of Geographic Sciences, East China Normal University, 200241 Shanghai, China; 11 Present Address: Environmental Research and Innovation Department, Luxembourg Institute of Science and Technology (LIST), L-4422 Belvaux, Luxembourg

## Abstract

Nitrous acid (HONO) is an important precursor of the hydroxyl radical (OH), the atmosphere´s primary oxidant. An unknown strong daytime source of HONO is required to explain measurements in ambient air. Emissions from soils are one of the potential sources. Ammonia-oxidizing bacteria (AOB) have been identified as possible producers of these HONO soil emissions. However, the mechanisms for production and release of HONO in soils are not fully understood. In this study, we used a dynamic soil-chamber system to provide direct evidence that gaseous emissions from nitrifying pure cultures contain hydroxylamine (NH_2_OH), which is subsequently converted to HONO in a heterogeneous reaction with water vapor on glass bead surfaces. In addition to different AOB species, we found release of HONO also in ammonia-oxidizing archaea (AOA), suggesting that these globally abundant microbes may also contribute to the formation of atmospheric HONO and consequently OH. Since biogenic NH_2_OH is formed by diverse organisms, such as AOB, AOA, methane-oxidizing bacteria, heterotrophic nitrifiers, and fungi, we argue that HONO emission from soil is not restricted to the nitrifying bacteria, but is also promoted by nitrifying members of the domains *Archaea* and *Eukarya*.

## Introduction

The photolysis of nitrous acid (HONO) yields the hydroxyl radical (OH), the primary oxidizing agent in the atmosphere, and thereby contributes significantly to the total daily primary OH production (up to 56%^1^), particularly in the extratropics. In numerous field studies, measured daytime mixing ratios of HONO have far exceeded model estimates based on known abiotic sources, such as gas phase formation from NO and OH and the heterogeneous disproportionation of NO_2_^2^. Consequently, several additional potential sources of HONO have been postulated^3^. Recently, soil was shown to be an important source of HONO due to the partitioning of nitrous acid between the aqueous phase of soil and the gas phase^4^. This physicochemical approach used the bulk nitrite concentration, the soil water content, and the pH-dependent equilibrium between HONO and nitrite to calculate the concentration of dissolved HONO in the aqueous phase of soil. By examining biological influences, Oswald *et al*.^5^ found strong HONO emissions mainly at low soil water content, with the highest emissions from soils at neutral pH, and identified ammonia-oxidizing bacteria (AOB) as a source of HONO. Indeed, *Nitrosomonas europaea* was shown to emit approximately four times more HONO than a sterile control^5^. A recent study successfully combined molecular biological surveys with isotopic measurements, to confirm AOBs as a source of soil emissions. It additionally revealed that more HONO is emitted with increasing soil pH^6^. Soil particle surfaces can be more acidic than the bulk pH, which might explain HONO release at higher bulk soil pH^7^. Interestingly, the optimum pH range of HONO emissions coincides with the optimum pH range for nitrification by AOBs^8,9^. Therefore, favorable conditions for microbial production (neutral-high pH) appear to be more important than favorable conditions for release (low pH). Despite these recent advances, and the insights in microbial pathways such as NH_2_OH oxidation to NO_2_^−^, the underlying mechanisms that govern the release and the precursor species for non-enzymatic HONO soil emissions remain largely unknown.

An essential and reactive intermediate of nitrification is hydroxylamine (NH_2_OH). This species was shown to decompose to N_2_O^10,11^, which has also been considered as a product of heterogeneous decomposition of HONO/NO_2_^−^ on soil surfaces^12,13^. In this study we investigate whether hydroxylamine decomposition can also form HONO. We used a dynamic soil-chamber method^14^ to investigate gaseous fluxes of NH_2_OH from pure cultures of AOB, nitrite-oxidizing bacteria (NOB), and the ammonia-oxidizing archaeon (AOA), *Nitrosopumilus maritimus*. The liquid culture suspension was applied to glass beads in a petri dish until water holding capacity (WHC) was reached. This setup simulates the soil matrix for the microbes and allows comparison with real soil. Release of gaseous NH_2_OH was measured by a Proton-Transfer-Reaction Time-of-Flight Mass Spectrometer (PTR-TOF-MS). The HONO formation from the reaction of gaseous NH_2_OH on glass beads was observed with a LOng Path Absorption Photometer (LOPAP).

## Results and Discussion

**Figure 1:**
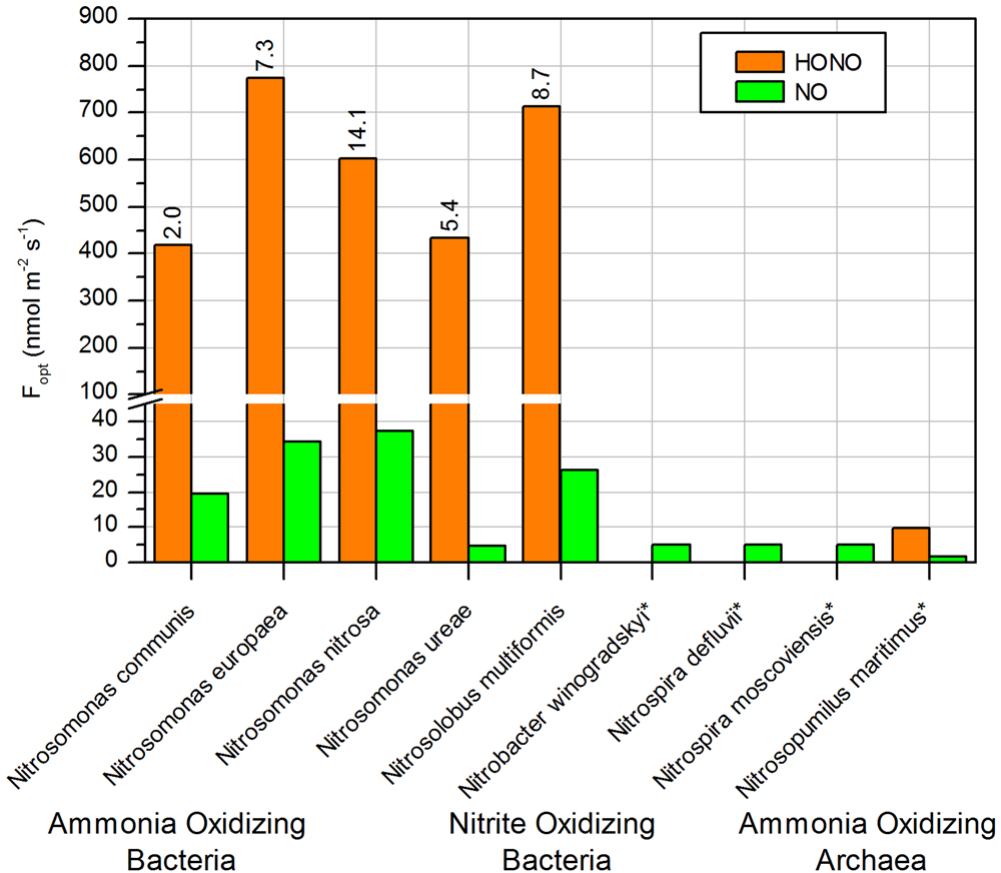
Optimum emissions (F_opt_) of HONO and NO from the investigated cultures of AOB, NOB and AOA. The cell densities of the pure culture expressed as ATP concentration in µmol l^−1^ are shown by the numbers above the corresponding bars. For NOB and AOA no ATP data is available. Measurements refer to single values as some cultures have been measured repeatedly but at different ATP concentrations (e.g. *N. europaeae* emitted 500 to 800 nmol m^−2^ s^−1^ for ATP between 2.5 µmol L^−1^ and 10 µmol L^−1^).

HONO and NO formation were investigated for *Nitrosomonas communis*, *Nitrosomonas europaea*, *Nitrosomonas nitrosa, Nitrosomonas ureae*, and *Nitrosolobus multiformis*, which represent all phylogenetic lineages comprising terrestrial and limnic AOB species^15^. Additionally, emissions from the NOB species *Nitrobacter winogradskyi*, *Nitrospira defluvii*, and *Nitrospira moscoviensis*, and the AOA *Nitrosopumilus maritimus*, which represents the first characterized AOA^16^, were measured. *N. maritimus* has been previously used as model organism to study the mechanism of ammonia oxidation in archaea^17–19^. Highest emissions of HONO and NO are typically found at a certain gravimetric soil water content (θ_g_), termed the optimal water content. These maximal emissions are henceforth denoted as F_opt_(HONO) and F_opt_(NO). All measured AOB and AOA strains emitted more HONO than NO (Fig. 1). These results are in good agreement with Oswald *et al*.^5^, who found HONO emissions to be three times higher than NO emissions for a culture of *N. europaea*. In contrast, the NOB, which served as a negative control, emitted only small amounts of NO and no emission of HONO was detectable. This was to be expected from their metabolism, but has not been shown experimentally before. Due to relatively low cell densities and slow growth rates, HONO emission from *N. maritimus* was low (See Fig. 1). Another reason for the low NH_2_OH release by AOA may be the nature of the membrane lipids and the thick protein layer surrounding the cells. The membrane consists of a monolayer of tightly packed etherlipids^20^ and the cell envelope is a hexagonally arrayed single S-layer^21^ that protect the cells from mechanical disruption^22^. Nevertheless, the measurements provided direct evidence for the potential of AOAs to contribute to HONO emissions. Like their bacterial counterparts, AOA generate energy by converting ammonia aerobically to nitrite via hydroxylamine^18^. The biochemical mechanism of ammonia oxidation in AOA, which is distinct from the bacterial pathway^19^, exhibits lower K_M_ values than those of AOB and enables AOA to thrive at extremely low ammonia concentrations^17^. Also in contrast to AOB, some AOA distantly related to *N. maritimus* are able to grow at low pH conditions. The obligate acidophilic AOA, *Nitrosotalea devanaerra*, was isolated from acidic soil and was found to be mainly responsible for autotrophic nitrification under acidic conditions^23^. Hence, we argue that globally abundant AOA in soils are likely responsible for biogenic HONO emissions at lower pH conditions than favorable for AOBs, as had been proposed by Scharko *et al*.^6^. This is supported by the results of Oswald *et al*.^5^ who investigated 17 soil samples and found an emission optimum at neutral pH that could be attributed to the activity of AOB. However, a second, smaller maximum was also found at a pH between 5 and 6. Under these conditions, even relatively acid-tolerant AOB are unlikely to be the source of the emissions^24^, but they appeared to be optimal for growth of the AOA, *N. devanaerra* (pH 5).

**Figure 2:**
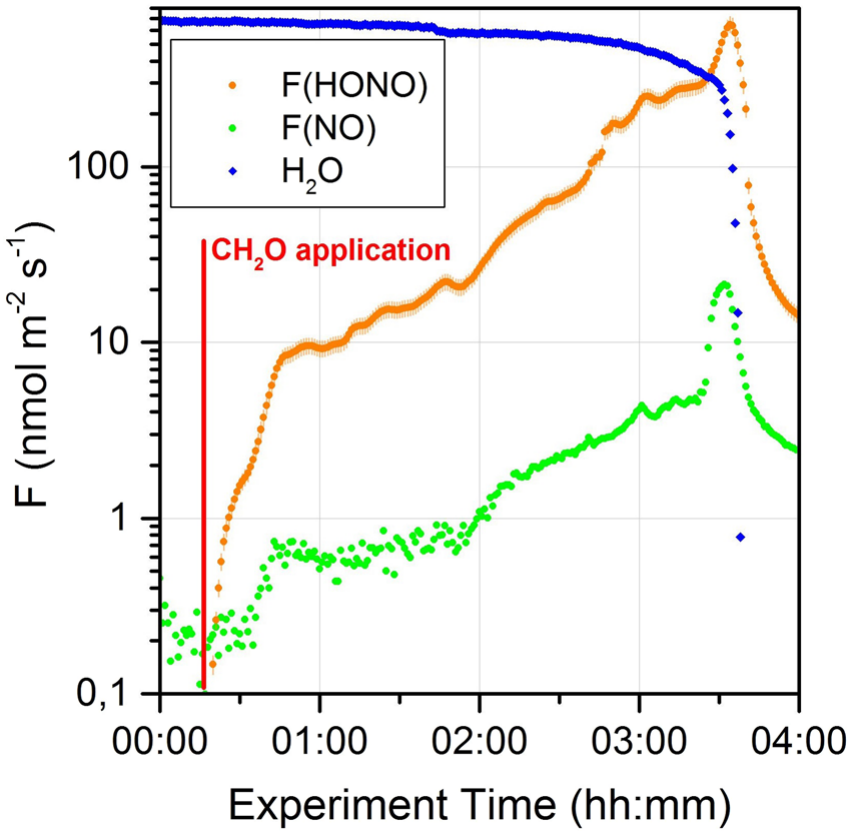
Increased porosity of the cell membrane of *N. europaea* by formaldehyde (CH_2_O) causes instantaneous release of HONO and NO. The release of H_2_O from the soil sample is represented in arbitrary units.

The energy metabolisms of both AOB^25^ and AOA^18^ use hydroxylamine (NH_2_OH) as an intermediate during the oxidation of ammonia (NH_3_) to NO_2_^−^. Comparison of the genomic inventory suggested that the responsible enzymes for both groups of ammonia-oxidizers differ strongly^19^, and so far the biochemical mechanism of the NH_2_OH to NO_2_^−^ conversion remains unknown for AOA^26^. Furthermore, the structures of the enzymes responsible for the reduction of NO_2_^−^ are different for AOA and AOB, and due to the marked differences in the enzymatic pathways of AOB and AOA, we focused our investigations on the metabolites that they have in common. Since we found no correlation between the NO_2_^−^ concentration in the culture solution and the release of HONO from cultures of *N. europaea* (Fig. S2), as would be expected from the partitioning of NO_2_^−^ ^4^, we focused on the intermediate, NH_2_OH. AOB are able to accumulate NH_2_OH to concentrations of up to 0.8 mol l^−1^ ^27^. In aqueous solutions, NH_2_OH is known to be autoxidized to NO_2_^−^ ^28,29^, providing a potential oxidation pathway to HONO. We added formaldehyde (CH_2_O) to a culture of *N. europaea* (Fig. 2) to increase the permeability of the cell membrane, and thereby to trigger the release of NH_2_OH and the formation of HONO. Release of HONO and NO occurred immediately after the addition of CH_2_O, suggesting that F(HONO) might be linked to the release of the internally accumulated precursor, NH_2_OH. In the study of Schmidt and coworkers^27^, 95% of the NH_2_OH inside AOB cells was found to be protein bound. As formaldehyde is known to denature proteins^30^ the applied CH_2_O may not only accelerate the release of internally accumulated NH_2_OH but also effectively release the bound NH_2_OH from the proteins and therefore accelerate the reaction. The release processes of NH_2_OH under natural conditions are still unknown. Stüven and coworkers proposed a pathway for NH_2_OH release to the surrounding media to explain NO and N_2_O formation by chemodenitrification^31^. The pathway that is utilized by AOB in suspension cultures (no drying out), involves additional electrons that originate from the oxidation of pyruvate or formate and cause an imbalance between ammonia and hydroxylamine oxidation, which leads to a release of NH_2_OH^31^. Furthermore, a recent study^32^ showed that *N. multiformis* and *N. europea* released NH_2_OH into the medium at measurable amounts, whereas NH_2_OH was not detectable in cultures of *N. nitrosa* and *N. communis* grown under the same conditions. Interestingly, in our experiments *N. multiformis* and *N. europea* showed larger HONO emissions than *N. nitrosa* and *N. communis* (see Fig. 1). Although the measurement of NH_2_OH in soils remains difficult due to its reactivity^12,33^, it is widely accepted that abiotic decomposition of NH_2_OH in soils is a non-negligible source of N_2_O from soils^11,12^. To further test if NH_2_OH is released by the microorganisms, we measured its concentration in the headspace gas of different soil samples.

**Figure 3:**
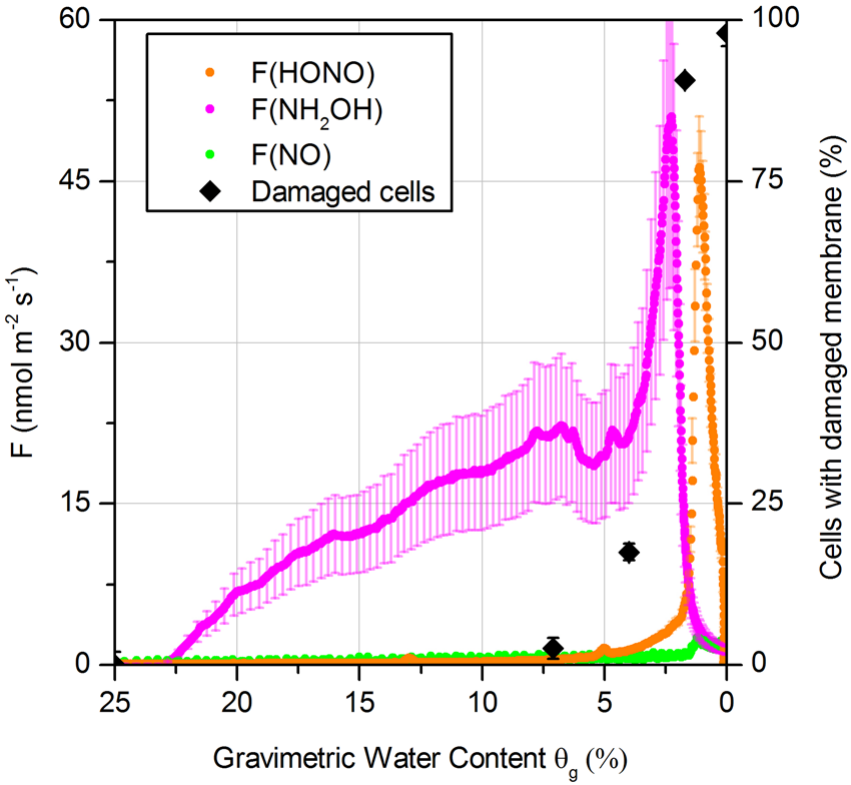
*N. europaea* releases NH_2_OH at the same order of magnitude as HONO, but it is emitted at higher gravimetric water content, θ_g_, compared to HONO. With decreasing θ_g_, the number of cells with membrane damage rises (the time course of the experiment proceeds from left to right). Error bars of damaged cell numbers denote standard deviations (n = 3).

A Proton-Transfer-Reaction Time-of-Flight Mass Spectrometer (PTR-TOF-MS) was used to confirm the release of NH_2_OH from a sample of an *N. europaea* culture, which had been applied to a glass bead matrix as described previously. In addition, we analyzed the number of membrane-damaged cells by fluorescence microscopy. As shown in Fig. 3, *N. europaea* released NH_2_OH during the experiment. The emission is observed over the entire soil moisture range, and a maximum is found at low θ_g_. This pattern differs from that of HONO, which is only released under dry conditions (θ_g_ < 3%). Most notably, the release of HONO rises at the same time as the NH_2_OH emission fluxes decline sharply. This strongly suggests a conversion of NH_2_OH to HONO at low θ_g_. The number of cells with a damaged membrane rises substantially at θ_g_ < 7% during the dry-out experiment. This temporal pattern matches with the increased release of NH_2_OH and the subsequent HONO formation from the *N. europaea* culture, supporting the hypothesis that accumulated NH_2_OH is released and acts as a precursor for the formation of HONO. Although the mechanism is not clear (see above), Fig. 3 shows that NH_2_OH is not only released by microorganisms under extreme conditions during dry-out finally causing cell death, but that NH_2_OH is released already at θg, <25%. Increasing numbers of damaged cells were only observed for θg, <7% accompanied with a sharp increase in NH_2_OH emissions (from ~20 nmol m^2^ s^−1^ to about 50 nmol m^2^ s^−1^), but already starting at a high level.

We further explored the potential conversion of NH_2_OH to HONO by investigating the heterogeneous reaction of NH_2_OH and water vapor to reflect the conditions at low θ_g_. We constructed a NH_2_OH permeation source, which supplied the experiment with 207 ppb of gaseous NH_2_OH in purified air. This gas stream was humidified and then passed through a cartridge with varying amounts of glass beads (i.e., varying the surface available for reaction) simulating the soil matrix.

**Figure 4:**
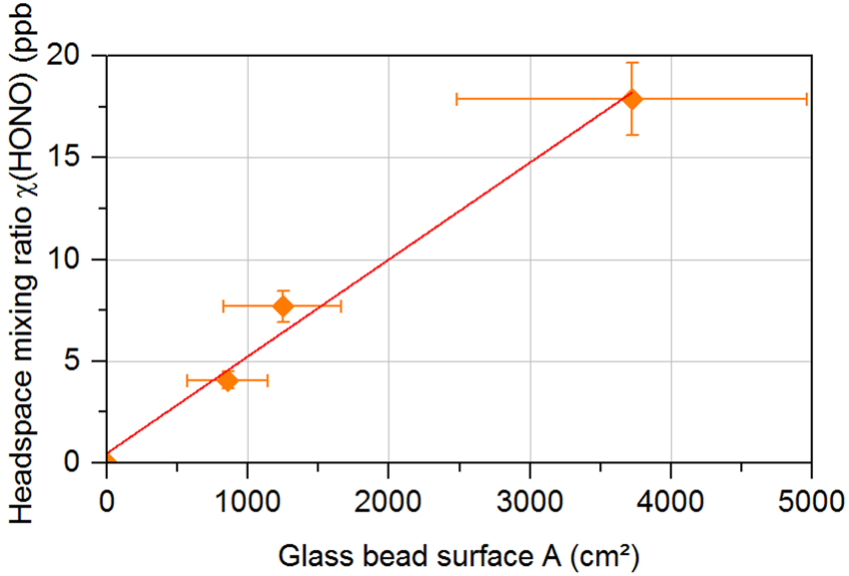
HONO is formed on glass bead surfaces during the surface reaction of NH_2_OH (207 ppb) with water vapor (12.5 mmol mol^−1^). The point at zero is the value measured without glass beads.

A linear relationship between the formed HONO and the glass bead surface area was found (Fig. 4) for constant levels of gaseous NH_2_OH. Therefore, the reaction can be summarized as:

R1 $${{\rm{NH}}}_{2}{\rm{OH}}+{{\rm{H}}}_{2}{\rm{O}}+{\rm{surface}}\to {\rm{HONO}}+{\rm{unknown}}\,{\rm{products}}$$

The highest conversion yield of NH_2_OH to HONO was 8.6%, with a contact time of ~3 s between the molecules and a glass bead surface of 3600 cm^2^. Under dry conditions (dew point of about −30 °C), no formation of HONO was observed demonstrating that water vapor is necessary for the reaction. Moreover, a significant gas phase reaction of NH_2_OH and water vapor was not found (measurement without glass beads Fig. 4). Our results therefore demonstrate that HONO is formed from NH_2_OH on surfaces at low humidity conditions. This heterogeneous reaction may explain why HONO emissions are restricted to low θ_g_ (<3%; see Fig. 3), as the glass bead surface is free of liquid water films, in which the slow autoxidation of NH_2_OH^28,29^ occurs, only under these dry conditions and is only then available for the surface reaction R1.

The analysis of four different soil samples did not show any measurable NH_2_OH emissions. It is possible that there was a small NH_2_OH release, which we were unable to detect because of the interference from the minor isotopomer of methanol (^13^CH_3_OH), as both signals overlap and are within the noise level of the instrument. However, we consider it more likely that the lack of observable NH_2_OH emission is explained by its efficient conversion to HONO in natural soils, since the specific surface area of natural soils (~10³–10^6^ cm² g^−1^) is significantly greater than that of the glass beads (59.6 cm² g^−1^)^34,35^. Our results support the assumption made by previous authors that NH_2_OH could not be detected in soils due to its reactivity^12,28^. By linearly extrapolating the relationship between surface area and conversion yield from our experiments (Fig. 4), a complete conversion can be expected under our experimental conditions (i.e., ~200 ppb NH_2_OH in air, residence time 3 sec) at around 4 × 10^4^ cm^2^, corresponding to only 0.4 g of a soil with a medium specific surface area of 10^5^ cm^2^ g^−1^. Furthermore, the decomposition of NH_2_OH and HONO/NO_2_^-^ is thought to be catalyzed by metal oxides^11,12,36^, many of which are present in soil. Consequently, the release of HONO by soil is partly attributable to NH_2_OH that has been converted within the soil matrix over the prolonged reaction time and high surface area. Therefore, NH_2_OH might also contribute indirectly to NO pulses observed during rewetting of the soils as they have been attributed to decomposition of HONO/NO_2_^−^, which had accumulated in the soil during dry-out (with a potential contribution from NH_2_OH decomposition), in their initial phase^37^.

## Conclusions

A new formation pathway for HONO in dry soils was identified. Our study reveals that NH_2_OH released by microorganisms, especially under the conditions of increased permeability of cell membranes or cell death during dry-out, is decomposed in soils and yields HONO by a heterogeneous reaction on soil particles involving NH_2_OH and H_2_O. This reaction may explain a substantial fraction of the HONO emission observed at low soil water content. Our results show that these biogenic HONO emissions are not restricted to AOB, but that also AOA are contributing to the release of this important trace gas.

Our results suggest that all organisms that produce NH_2_OH at some stage in their metabolism are potential contributors to the formation of HONO. Within the nitrogen cycle, NH_2_OH is also produced during the heterotrophic nitrification from bacteria of different genera^38^. Methane-oxidizing bacteria are another group of bacteria that is capable of producing NH_2_OH^39^, and even eukaryotic species such as the fungus *Aspergillus flavus* have been shown to be a source^40^. Hence, the capability to contribute to HONO formation in soils is present in all three domains of life, including bacteria, archaea, and eukaryotes.

## Methods

We used the dynamic chamber method^14^ (and below) to investigate gaseous fluxes from pure cultures of AOB, nitrite-oxidizing bacteria (NOB) and the ammonia-oxidizing archaeon (AOA), *Nitrosopumilus maritimus*. Liquid culture suspension from pure cultures of AOB, nitrite-oxidizing bacteria (NOB) and the ammonia-oxidizing archaeon (AOA), *Nitrosopumilus maritimus*, were applied to soda lime glass beads (0.25–0.50 mm diameter, Carl Roth, Germany) in a petri dish until water holding capacity (WHC) was reached. This setup simulates the soil matrix for the microbes and allows comparison with real soil. The petri dish containing the sample was subsequently placed into the dynamic chamber, which was flushed with purified dry air (free from NO, NO_2_, O_3_, H_2_O, VOCs, NH_2_OH, and HONO). Due to the flow of dry air over the sample, it is gradually dried out in the course of an experiment. The cell density of a culture was characterized by the content of adenosine 5′-triphosphate (ATP) (µmol l^−1^), which correlated well with the microscopically derived cell density (Fig. S1). Prior to each experiment, the cell density was determined and found to be between 2 to 20 µmol l^−1^ ATP (data not available for NOB and AOA experiments). Significant growth of the culture during an experiment can be excluded, as typical doubling times of AOB are between 12 and 20 hours^41^, and ≥21 hours for the investigated AOA^16^, whereas the duration of an experiment was 6 to 10 hours.

### Dynamic Chamber

A chamber coated with polyfluoroethylene (PFE) foil with a volume of 0.008 m³ was flushed at a flow rate of 1·10^−4^ m^3^ s^−1^ with dry purified air (Figure S3). Air purification was achieved as follows: the air was passed through a membrane dryer combined with a filter for compressed air (Clearpoint and Drypoint M from BEKO Deutschland GmbH, Germany). In a second step, a UV lamp (OG-1, Ultra-Violet Products Ltd, USA) was used to photolyze HONO to NO and OH. A pure-air generator (PAG 003, ECOPHYSICS, Switzerland) was used to remove further trace gases such as HONO, NO_x_, O_3_, hydrocarbons, and water vapor. To prevent any traces of reactive nitrogen gases from entering the system, a cartridge filled with Purafil (Headline Filters, Germany) was installed after the pure air generator. To ensure sterile conditions in the chamber, the inlet was equipped with a sterile air filter (MILEX®-FG Vent Filter 0.2 µm, 50 mm diameter, Millipore, France). NO_x_ (NO + NO_2_) was measured at the outlet of the chamber by a chemiluminescence analyzer (CLD 780TR, ECOPHYSICS, Switzerland, limit of detection (LOD): LOD_NO_ ≈ 35 ppt and LOD_NO2_ ≈ 120 ppt), where NO_2_ was detected after conversion to NO by a blue light converter (Air Quality Design, Inc., Co, USA). A UV-absorption analyzer (Model 49i, Thermo Electron Corporation, USA; LOD ≈ 0.5 ppb) was used to measure O_3_ levels to ensure that no outside air was entering the system^.^ The water vapor difference between the inlet and outlet of the chamber was measured with an infrared gas analyzer (LI-7000, Li-Cor Biosciences GmbH, Germany). To avoid any wall losses, the long path absorption photometer (LOPAP) (QUMA Elektronik & Analytik GmbH, Wuppertal, LOD ≈ 5 ppt) was directly connected to the chamber to measure HONO. The chamber and the LOPAP sampling unit were placed in a temperature-controlled cabinet. Data were acquired by a CR3000 data logger (Campbell Scientific, Inc., USA) every 60 s.

### NH_2_OH measurements

Gaseous hydroxylamine (NH_2_OH) was determined with a commercial PTR-TOF-MS (Proton-Transfer-Reaction Time-of-Flight Mass Spectrometer, Ionicon Analytik GmbH, Innsbruck, Austria)^42^. This measurement technique utilizes the protonation of molecules with a proton affinity higher than water by H_3_O^+^ ions that are generated in a hollow cathode discharge. NH_2_OH has a proton affinity of 803 kJ mol^−1 ^^43^, while the value for water is 691 kJ mol^−1 ^^44^. All protonated molecular ions are accelerated by an electrical field to the same kinetic energy such that the resultant velocity of the ions depends on the mass-to-charge ratio. Hence, the velocity is measured as the time-of-flight, from which the mass-to-charge ratio can be calculated^45^. The mass resolution was approximately 3700 m/∆m and NH_2_OH was measured at mass 34.029. It should be noted that the ^13^C isotope of methanol at mass 34.037, which represents 1% of the methanol signal, potentially interferes with ambient NH_2_OH measurements from soil, but not with those from the cultures. For the experiment with a pure culture of *N. europaea*, NH_2_OH was in large excess over methanol. The instrument was operated with a drift pressure of 2.20 hPa (E/N 140 Td) and a drift voltage of 600 V. 1,3,5-trichlorobenzene was used as internal standard for mass scale calibration. Data post-processing and analysis was performed by using the program “PTR-TOF DATA ANALYZER”^46^. The NH_2_OH measurements were made with the chamber system described by Behrendt *et al*.^47^. The effect of wall losses was minimized by using the same inlet tube length for calibration and measurement. The instrument was calibrated for gas phase NH_2_OH using a custom built sublimation unit with NH_2_OH purified by the method of Chang *et al*.^48^. NH_2_OH was exposed to a nitrogen gas flow and the concentration was determined gravimetrically. The PTR-TOF-MS was calibrated with a NH_2_OH mixing ratio of 893.8 ppb. Since the measured mixing ratios of NH_2_OH were lower (0–230 ppb) than the single-point calibration value, we assume a systematic error of 30%, in contrast to compounds calibrated with pressurized gas standards, which typically have an overall uncertainty of about 10%. The calculated detection limit (3σ of the noise) was about 15 ppt. For the surface reaction experiment with glass beads, the gas flow from the NH_2_OH source (745 ppb) was diluted with humidified air (12.5 mmol mol^−1^ H_2_O) to a mixing ratio of 207 ppb NH_2_OH and directly passed through a cartridge filled with the glass beads. The HONO produced from wall reactions inside the system (without glass beads) has been subtracted.

The measurement of NH_2_OH by any technique requiring tubing connections is challenging, because this molecule has a high affinity to adsorb on tubing walls due to its polarity. The possibility that the maximum F(NH_2_OH) observed in Fig. 3 was related to a desorption of NH_2_OH from the tubing, caused by the decreasing humidity, is unlikely, since similarly soluble molecules, e.g., methanol, did not show such effects. Based on humidity dependent calibrations of multiple volatile organic compound standards, the peak of NH_2_OH shown in Fig. 3 is far too large to be explained by instrumental sensitivity changes associated with humidity. Extensive calibrations with the analogous species methanol between 25–80% RH have shown variations of between 10–20% in sensitivity, whereas many compounds such as isoprene show no humidity dependence at all.

Further information on cultivation of microorganisms, soil sampling and analysis, measurement of microorganisms and soil samples, and flux calculations can be found in the supplementary information.

### Data availability

Data are available on request from m.soergel@mpic.de.

## Electronic supplementary material

Supplementary information
