# Supplementary material for: Hydroxylamine released by nitrifying microorganisms is a precursor for HONO emission from drying soils
Source: Sci Rep. 2018 Jan 30;8:1877. doi: 10.1038/s41598-018-20170-1 (PMC5790002; doi:10.1038/s41598-018-20170-1)
Supplement: Supplementary file 1 — Supplementary information [file 41598_2018_20170_MOESM1_ESM.pdf]

Title: Hydroxylamine released by nitrifying microorganisms is a precursor for HONO emission from drying soils

Authors: M. Ermel[1,2,#], T. Behrendt[1,3], R. Oswald[1], B. Derstroff[4], D. Wu[1,5,†], S. Hohlmann[1], C. Stönner[4], A. Pommerening-Röser[6], M. Könneke[7], J. Williams[4], F. X. Meixner[1], M. O. Andreae[1,8], I. Trebs[1,+] and M. Sörgel[1,*]

Author Affiliations:

[1] Biogeochemistry Department, Max Planck Institute for Chemistry, P.O. Box 3060, 55020 Mainz, Germany

[2] Institute for Inorganic and Analytical Chemistry, Johannes Gutenberg University Mainz, 55128 Mainz, Germany

[3] Biogeochemical Processes Department, Max Planck Institute for Biogeochemistry, P.O. Box 10 01 64, 07745 Jena, Germany

[4] Atmospheric Chemistry Department, Max Planck Institute for Chemistry, P.O. Box 3060, 55020 Mainz, Germany

[5] Key Laboratory of Agricultural Water Research, Center for Agricultural Resources Research, Institute of Genetic and Developmental Biology, The Chinese Academy of Sciences, 050021 Shijiazhuang, China

[6] Department of Microbiology and Biotechnology, University of Hamburg, Hamburg, Germany

[7] MARUM Center for Marine Environmental Sciences and Department of Geoscience, University of Bremen, P.O. Box 330440, 28334 Bremen, Germany

[8] Scripps Institution of Oceanography, University of California San Diego, San Diego, CA 92093

#now at: Messer Industriegase GmbH, Messer-Platz 1, 65812 Bad Soden, Germany

†now at: Key Laboratory of Geographic Information Sciences, Ministry of Education, School of Geographic Sciences, East China Normal University, 200241 Shanghai, China

[+]now at: Environmental Research and Innovation Department, Luxembourg Institute of Science and Technology (LIST), L-4422 Belvaux, Luxembourg

***Corresponding Author:** Matthias Sörgel (Max Planck Institute for Chemistry, P.O. Box 3060, 55020 Mainz, m.soergel@mpic.de, +4961313054432)

Materials and methods

Cultivation of bacteria. Ammonia-oxidizing bacteria were cultivated in a culture suspension containing 10 mM NH_4^+ according to Krümmel and Harms [1]. The cultures were checked for their purity microscopically and by the use of a nutrient broth test (Standard 1 nutrient broth, Merck KGaA, Germany), which ensures that the cultures were free of heterotrophic contaminants[2]. The following strains of AOB, NOB, and AOA were used in this study: *Nitrosomonas communis* (Nm2)[3], *Nitrosomonas europaea* (Nm50, ATCC 25978)[4], *Nitrosomonas nitrosa* (Nm90)[3], *Nitrosomonas ureae* (Nm10)[3], *Nitrosolobus multiformis* (Nl13)[5], *Nitrobacter winogradskyi* (« Engel »), *Nitrospira defluvii* (A17), *Nitrospira moscoviensis* (M), and N*itrosopumilus maritimus*[6]

Soil sampling and analysis. The soil samples were taken from a wheat field (Mainz-Finthen, Germany, 49.97°N, 8.16°E)[7]. The uppermost soil layer (5 cm) was used for the experiments. The sample was dried at 40 °C for 24 hours, sieved to 2 mm, and stored at 4 °C in open plastic bags before measurement.

Measurement of bacteria and soil samples. All samples were prepared in petri dishes (100 x 20 mm, Duran Group, Germany) with 50 g of either soil sample or glass beads (0.25 – 0.50 mm diameter, Carl Roth, Germany)[7]. For NH_2OH measurements, a smaller petri dish (50 x 20 mm, Duran Group, Germany) with 12 g of glass beads and soil, respectively, was used. The soil samples were wetted with purified water to reach water holding capacity (WHC), see Behrendt et al.[8]. The sample was subsequently placed into the dynamic chamber.

For bacteria culture samples, the glass beads and glass bowl were sterilized by washing with 70 % ethanol (absolute for analysis, Merck, Germany). Sterility of the setup was checked by an ATP assay on a sample of sterile AOB nutrient solution. Bacteria culture suspension or sterile culture solution was added to the glass beads to reach WHC and the sample was subsequently

placed into the dynamic chamber. Prior to the measurement, the cell density of the culture was measured using an ATP kit (BacTiterGlo, PROMEGA GmbH, Germany) and a luminometer (GloMax 20/20, PROMEGA GmbH, Germany). The NO_2^- concentration in the culture suspension was measured according to ISO/TS 14256-1. The NO_2^- from this reaction was also determined according to ISO/TS 14256-1.

Flux calculations. HONO, NH_2OH and NO fluxes were calculated according to the following formula:

$$F = \frac{Q}{A \cdot V_m} \cdot (\chi_{out} - \chi_{in}) \qquad (2)$$

where F is the flux of trace gas in nmol m^{-2} s^{-1}, Q is the purging flow rate in m^3 s^{-1}, A is the area of soil in m^2, V_m is the molar volume of air in m^3 mol^{-1}, and χ_{out} and χ_{in} are the headspace mixing ratios at the outlet and inlet of the chamber, respectively, in ppb.

The error of F was calculated as follows:

$$\Delta F = \pm\sqrt{\left(\left[\left(\frac{\partial F}{\partial Q}\right)_{A,\chi_{in/out}} \cdot \Delta Q\right]^2 + \left[\left(\frac{\partial F}{\partial A}\right)_{Q,\chi_{in/out}} \cdot \Delta A\right]^2 + \left[\left(\frac{\partial F}{\partial \chi}\right)_{A,Q,\chi_{in}} \cdot \Delta \chi_{out}\right]^2 + \left[\left(\frac{\partial F}{\partial \chi}\right)_{A,Q,\chi_{out}} \cdot \Delta \chi_{in}\right]^2\right)} \qquad (3)$$

The error of V_m was neglected. The error of A was assumed to result from a 1 mm uncertainty of the dish radius. The noise (3σ) of the measured flow rate was used to estimate ΔQ. The error of χ_{in} was set to the limit of detection of the instruments. For the error of χ_{out} of HONO, an additional uncertainty of 10 % of the absolute value was added to the limit of detection. For the other species the error of χ_{out} was derived as for χ_{in}.

Supporting Figures

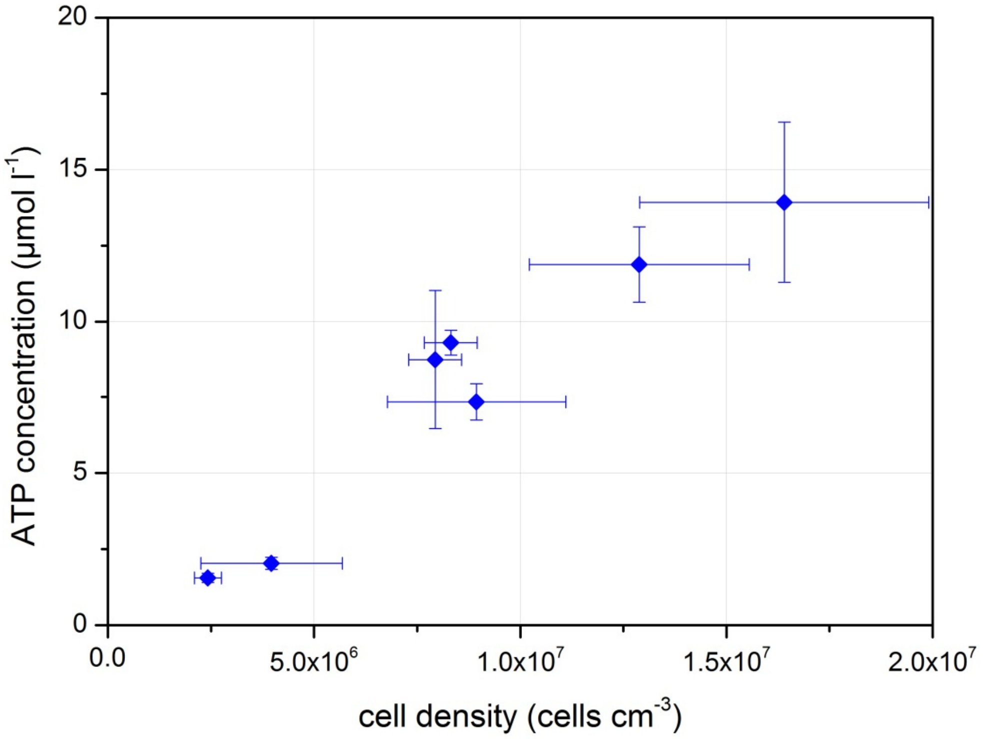

Figure S1: The ATP concentration of a culture as a function of the microscopically determined cell density. Error bars denote standard deviations (n=3).

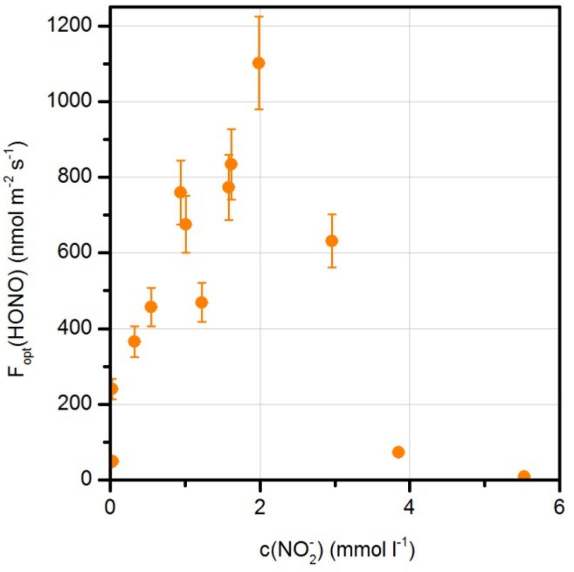

Figure S2: Optimum emissions, F_{opt}(HONO), for *Nitrosomonas europaea* cultures as a function of NO_2^- concentration in the culture.

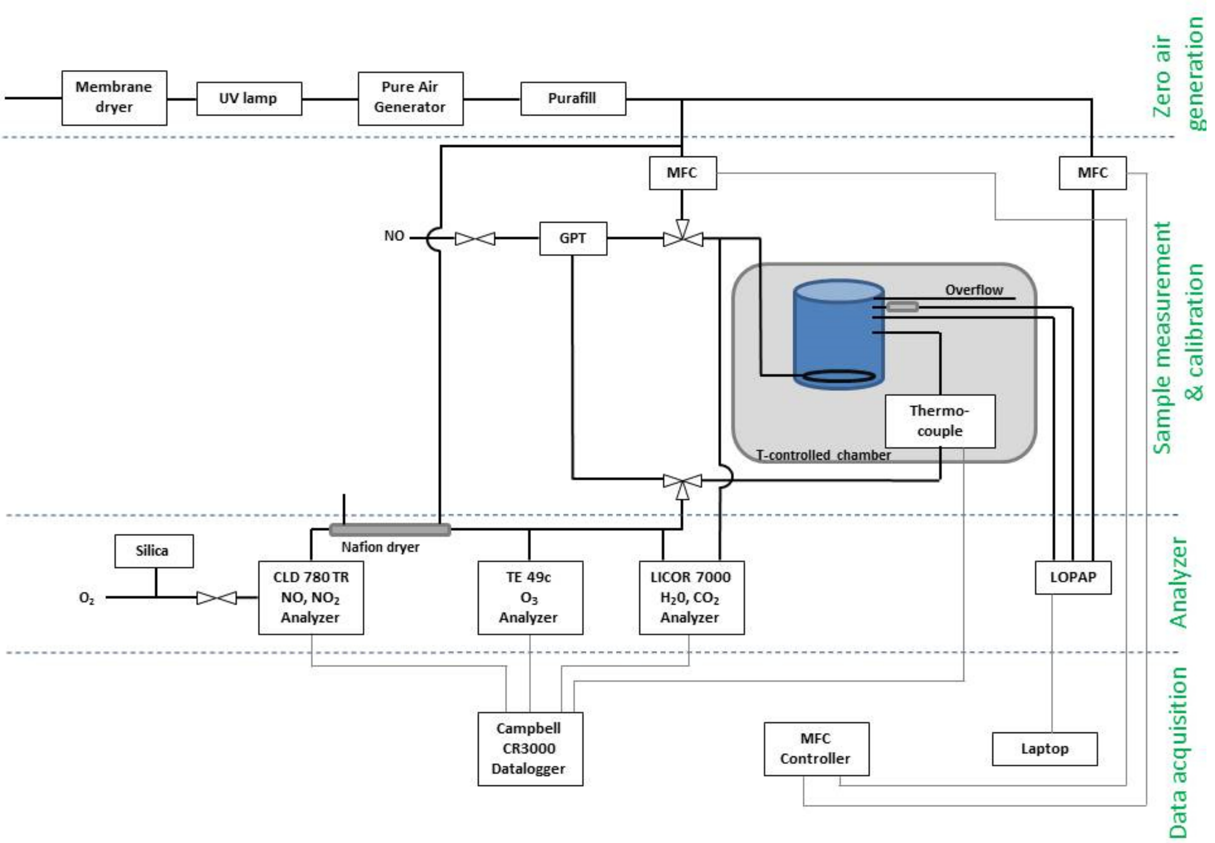

Figure S3: Schematic overview of the setup used to measure F(HONO) and F(NO). It can be separated into zero air generation, measurement and calibration, analyzer, and data acquisition.

References

1 Krümmel, A. & Harms, H. Effect of organic matter on growth and cell yield of ammonia-oxidizing bacteria. Arch. Microbiol. **133,** 50-54, doi:10.1007/bf00943769 (1982).

2 Koops, H. P., Harms, H. & Wehrmann, H. Isolation of a Moderate Halophilic Ammonia-Oxidizing Bacterium, Nitrosococcus mobilis Nov Sp. Arch. Microbiol. **107,** 277-282, doi:Doi 10.1007/Bf00425339 (1976).

3 Koops, H. P., Böttcher, B., Möller, U. C., Pommerening-Röser, A. & Stehr, G. Classification of 8 New Species of Ammonia-Oxidizing Bacteria - Nitrosomonas communis Sp nov, Nitrosomonas ureae Sp nov, Nitrosomonas aestuarii Sp nov, Nitrosomonas marina Sp nov, Nitrosomonas nitrosa Sp nov, Nitrosomonas eutropha Sp nov, Nitrosomonas oligotropha Sp nov and Nitrosomonas halophila Sp nov. J. Gen. Microbiol. **137,** 1689-1699 (1991).

4 Watson, S. W. Taxonomic considerations of the family Nitrobacteraceae Buchanan requests for opinions. Int. J. Syst. Bacteriol. **21,** 254-270 (1971).

5 Watson, S. W., Graham, L. B., Remsen, C. C. & Valois, F. W. Lobular, Ammonia-Oxidizing Bacterium, Nitrosolobus multiformis Nov Gen Nov Sp. Arch. Mikrobiol. **76,** 183-&, doi:Doi 10.1007/Bf00409115 (1971).
6 Könneke, M. et al. Isolation of an autotrophic ammonia-oxidizing marine archaeon. Nature **437,** 543-546, doi:Doi 10.1038/Nature03911 (2005).
7 Oswald, R. et al. HONO Emissions from Soil Bacteria as a Major Source of Atmospheric Reactive Nitrogen. Science **341,** 1233-1235, doi:10.1126/science.1242266 (2013).
8 Behrendt, T. et al. Characterisation of NO production and consumption: new insights by an improved laboratory dynamic chamber technique. Biogeosciences **11,** 5463-5492, doi:10.5194/bg-11-5463-2014 (2014).
